# Vitamin D status and surgical outcomes: a systematic review

**DOI:** 10.1186/s13037-015-0060-y

**Published:** 2015-04-30

**Authors:** Paul J Iglar, Kirk J Hogan

**Affiliations:** Department of Population Health Sciences, University of Wisconsin School of Medicine and Public Health, 707 WARF Building, 610 North Walnut Street, Madison, WI 53726 USA; Department of Anesthesiology, University of Wisconsin School of Medicine and Public Health, 600 Highland Avenue, B/6 319 Clinical Sciences Center, Madison, WI 53792-3272 USA

**Keywords:** Vitamin D, 25(OH)D (calcifediol), 25(OH)D (calcifediol) level, 1,25-dihydroxyvitamin D3 (calcitriol), 1,25-dihydroxyvitamin D3 (calcitriol) level, Surgery outcome, Surgery complication, Postoperative outcome, Postoperative complication

## Abstract

The importance of vitamin D for musculoskeletal health has long been recognized, and awareness of significant extra-skeletal effects in health and disease is rapidly emerging. Although it has been possible for many decades to quantify serum markers of vitamin D deficiency, and to correct deficiency at low cost and with high safety, the influence of vitamin D status on post-surgical outcomes has only recently been identified as a research topic of interest. To the present, these data have not been the subject matter of formal review. Accordingly, we conducted a systematic review to assess the association between perioperative vitamin D status and outcomes after surgery. The databases of PubMed, Ovid MEDLINE, EMBASE, AMED, CINAHL (EBSCOHost), The Cochrane Databases of Systematic Review, and PROSPERO were searched through December, 2014 for studies relating to vitamin D and surgery. The initial search yielded 90 manuscripts. After applying exclusion criteria, 31 studies were eligible for inclusion. Fifteen studies employed prospective observational designs, 3 used prospective randomized protocols, and 13 report retrospective database interrogations. The main finding of the present review is that 26 of 31 studies (84%) report at least one statistically significant worse outcome in patients with low vitamin D status. Five of 31 studies (16%) found no association. In conclusion, this review supports the hypothesis that hypovitaminosis D is associated with adverse outcomes after diverse surgical procedures. Future studies should focus on additional surgeries and outcomes, and on the role of vitamin D supplementation in the improvement of patient safety in participants with low vitamin D status at the time of surgery.

## Introduction

Vitamin D, a fat soluble steroid hormone formed primarily by photosynthesis, plays a key role in over 300 metabolic pathways in humans through a specific nuclear-binding receptor, and mechanisms for signal transduction that are expressed in most cells and tissues [1,2]. Although the importance of vitamin D for musculoskeletal health has long been known, awareness of significant extra-skeletal effects in health and disease is rapidly emerging [3,4]. Deleterious effects of vitamin D deficiency on medical and surgical critical care outcomes have recently been reviewed, and striking improvements in intensive care mortality after vitamin D replacement therapy have been reported [5,6]. It has been possible for many decades to quantify serum markers of vitamin D deficiency, and to correct deficiency at low cost and with high safety, however, the influence of vitamin D status on post-surgical outcomes is less well-recognized [3,4]. Whereas critical care outcomes have generally been reported in a shared core of journals, post-surgical outcomes appear in diverse, specialty-oriented publications that may be less likely to cross-reference one another (see below). Vitamin D deficiency is defined by the Institute of Medicine (IOM) as a 25(OH)D of less than 20 ng/ml [3]. Vitamin D insufficiency is defined as a 25(OH)D of 21–29 ng/ml [3]. In keeping with these definitions, the estimated prevalence of vitamin D insufficiency is as high as 50 to 80% in the general population [4]. Children, young adults, middle-aged adults and elderly adults are at comparably high risk for vitamin D deficiency and insufficiency [4]. Accordingly, it is the aim of the present systematic review to gather and evaluate published data that correlates perioperative vitamin D status with post-surgical outcomes for analytical validity, clinical validity and clinical utility, and to identify research priorities with the potential to streamline their impact on public health and patient safety.

## Methods

### Inclusion criteria

A systematic review to detect published relationships in the peer-reviewed literature between serum vitamin D status and post-surgical outcomes was conducted. The study protocol was developed to ascertain both observational studies and controlled trials that compare one or more serum indices of vitamin D status with any adverse outcome after surgery. The primary inclusion criteria comprised measurement of serum 25-hydroxy vitamin D (25(OH)D) and/or 1,25-dihydroxyvitamin D (1,25(OH)2 D) in the interval spanning 12 weeks before to 12 weeks after any type surgery. This time frame was chosen in keeping with the stability and kinetics of serum 25(OH)D, the most widely used circulating vitamin D biomarker, over time [7]. Any measure of a postoperative outcome or complication was accepted. Outcomes directly related to the target tissue of surgery (*e.g.*, surgical site infection), and those related to tissues other than the target tissue of surgery (*e.g.*, hospital-acquired infection, in-hospital mortality) were incorporated into the search. Secondary outcomes were also reviewed. Adult and pediatric investigations with female and male participants of any age qualified for inclusion. The study protocol was developed to review both retrospective and observational prospective (cross-sectional, case–control and cohort) study designs, and interventional studies, both randomized and non-randomized, as long as serum vitamin D status was reported before and after treatment and surgery. Because the entire evidence database was required to address the aims of the review, studies were not excluded on the basis of their methodological quality or sample size. Publication dates were not restricted.

### Exclusion criteria

Case reports, letters, stand-alone abstracts, narrative reviews, animal studies, duplicates, editorials and articles not available in full English translation were excluded. Investigations reporting only the prevalence of sub-threshold values before surgery that were not tested for correlation to surgical outcomes were omitted, although citation to these references are frequently cited in the manuscripts reviewed herein. Studies that correlated preoperative vitamin D status with other pre-operative values and pathologies in the absence of post-operative outcomes, or that correlate vitamin D status with increased need for surgery independent of adverse post-surgical events, were similarly eliminated from review. Manuscripts describing the direct effects of surgery on serum vitamin D levels (*e.g.*, vitamin D status as an outcome after renal transplantation and gastric surgery), and hypocalcemia after parathyroid and thyroid surgery (*e.g.*, secondary hyperparathyroidism after renal transplantation) were not considered.

### Search strategy

Potentially relevant articles were identified by a comprehensive search of the peer-reviewed literature in publicly available computerized databases. Electronic databases searched were: PubMed, Ovid MEDLINE, EMBASE, AMED, CINAHL (EBSCOHost), The Cochrane Databases of Systematic Review, and PROSPERO. The search was performed with the assistance of a librarian experienced in systematic reviews following PRISMA (Preferred Reporting Items for Systematic Reviews and Meta-Analyses) statement guidelines, a checklist and phase flow diagram constructed to enhance the quality of systematic reviews [8]. A structured search strategy for MEDLINE was based on a controlled vocabulary and relevant key terms configured for breadth to prioritize sensitivity. Manuscripts were identified using one, or a Boolean combination of two or more, of the following search terms: “vitamin D”; “25(OH)D (calcifediol)”; “25(OH)D (calcifediol) level”; “1,25-dihydroxyvitamin D3 (calcitriol)”; “1,25-dihydroxyvitamin D3 (calcitriol) level”; “surgery outcome”; “surgery complication”; “postoperative outcome”; “postoperative complication”; and diverse surgical procedures. These terms were then adapted for other databases. Initial search results were followed by targeted author searches to detect additional sources. References of included manuscripts and accompanying editorials, bibliographies of review articles, letters to editors and author responses to primary sources, and clinical trial registries were scanned for further citations. Included manuscripts were searched using the Related Articles feature in PubMed, Cited Reference Search in ISI Web of Science, and Cited By and Related Articles features of Google to identify further references. The title and abstract of each citation was reviewed by two independent reviewers, and potentially eligible articles were retrieved for scrutiny in full text. Discrepancies in eligibility between reviewers were resolved by consensus. The initial search was done from June, 2014 to December, 2014.

### Data extraction

Standardized data extracted from each publication that met inclusion criteria consisted of: PubMed ID number; author listing; journal; year of publication; number and location of participating centers; participant number, gender, age, and type of surgery of the population sample; type of study and design; inclusion and exclusion criteria; date of vitamin D status testing vs. date of surgery; marker of vitamin D status that was measured; vitamin D analysis method; vitamin D status reporting and statistical analysis methods (*i.e.,* as a categorical variable by threshold, quintiles, or as a continuous variable); specific primary and secondary outcomes sought; methods of scoring outcomes; time frame for scoring outcomes after surgery and the length of clinical follow up; presence or absence of power calculation for a primary outcome; methods of descriptive and inferential statistical analysis; presence or absence of at least one multivariate analysis; significant positive and negative correlations of perioperative vitamin D status and observed primary and secondary outcomes; effect size; and study strengths, weaknesses, and gaps. The extracted data was verified by a second reviewer to reduce errors and bias.

## Results

**Figure 1:**
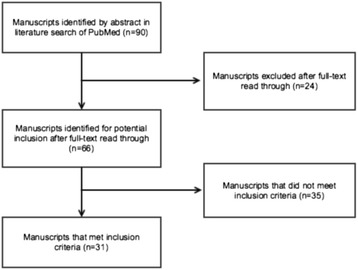
Diagram of the manuscript selection process from database search to inclusion.

The titles and abstracts of 90 manuscripts were found to potentially fulfill search criteria. After application of inclusion standards and full text review, 31 manuscripts remained for inclusion in 28 different journals (Figure 1). Table 1 provides summary data of retrieved manuscripts grouped by the type of surgery and by the date of publication. Of 31 manuscripts meeting inclusion criteria, 15 used prospective observational designs, 3 applied prospective randomized protocols, and 13 report retrospective interrogations of databases. In aggregate, post-surgical outcomes were scored in 16,195 individuals with study population sample sizes varying from 18 to 4,418 patients. The main finding of the present review is that 26 of 31 publications report at least one statistically significant correlation between low perioperative vitamin D status and at least one deleterious postoperative outcome. In most accounts, the clinical significance of low perioperative vitamin D status is substantial. For example, Ducloux *et al*. observed an 8% increased risk of cancer after kidney transplantation for each 1 ng/mL decrease in serum 25(OH)D, and a 4-fold greater risk of cancer overall in patients with low vitamin D status [22]. Falkiewiscz *et al*. found an 8-fold greater risk of delayed renal graft failure with perioperative vitamin D deficiency [23]. Patients with preoperative serum 25(OH)D less than 30 ng/mL were at 3- to 4-fold greater risk for hospital-acquired and surgical site infections in data analyzed by Quraishi *et al*. [21]. Low vitamin D status in the near transplant interval predicts a 3-fold greater risk of moderate to high grade organ rejection and 5-fold greater mortality in the first year after pulmonary transplantation in patients investigated by Lowery *et al*. [28].

**Table 1:** **Summary data of retrieved manuscripts grouped by the type of surgery and by the date of publication**

**Reference (Ref. No.)**	**Surgery**	**Design**	**Mean Age**	**Sample Size**	**Power analysis**	**Assay**	**Categorical or Continuous**	**Duration**	**Adverse Postoperative Outcomes Associated with Low Vitamin D Status**
Nawabi *et al*. [9]	hip arthroplasty	prospective, observational	71	62	no	25(OH)D, RIA	categorical, 16 ng/mL threshold	6 months	decreased Harris hip scores
Unnanuntana *et al.* [10]	hip arthroplasty	retrospective	66	200	no	25(OH)D, RIA	categorical, < 20, 20–31, > 31 ng/mL thresholds	to discharge	decreased ambulation distance after surgery
Unnanuntana *et al.* [11]	hip arthroplasty	prospective, observational	67	178	yes	25(OH)D, RIA	categorical, 30 ng/mL threshold	6 weeks	none
Lavernia, *et al.* [12]	hip arthroplasty	retrospective	70	60	no	25(OH)D, HPLC	categorical, 20 ng/mL and 30 ng/mL thresholds compared	3-24 months	decreased Harris and Merle d’Aubigne-Postel hip scores
Mak *et al.* [13]	hip fracture surgery	prospective, randomized trial	84	218	no	25(OH)D, RIA	continuous	26 weeks	increased pain scores
Reid *et al*. [14]	knee arthroplasty	prospective, observational	75	33	no	25(OH)D, LC-MS/MS	continuous	3 months	vitamin D status declines for 3 months after surgery, is not correlated with changes in C-reactive protein concentrations
Jansen *et al*. [15]	knee arthroplasty	prospective, observational	71	139	no	25(OH)D, RIA	categorical, 16 ng/mL threshold	6 months	decreased Knee Society scores, “lack of power”
Barker *et al*. [16]	anterior cruciate ligament repair	retrospective	31	18	no	25(OH)D, CIA	categorical, 30 ng/mL threshold	3 months	decreased postoperative knee strength
Lee *et al*. [17]	wrist fracture surgery	retrospective	>50	63	yes	25(OH)D, LC-MS/MS	categorical, < 20, 20–32, > 32 ng/mL thresholds	6 months	recovery of grip strength correlates with vitamin D supplementation, not baseline vitamin D status
Maier *et al*. [18]	hip, knee and shoulder prosthesis	prospective,observational	67	190	no	25(OH)D, CIA	categorical, 30 ng/mL threshold	variable	periprosthetic joint infection
Kim *et al*. [19]	spinal fusion	prospective, observational	66	31	no	25(OH)D, RIA	categorical, <20, 20–30, > 30 ng/mL thresholds	12 months	decreased Oswestry Disability Index and quality of life scores
Carlin *et al*. [20]	gastric bypass	prospective, randomized trial	43	60	no	25(OH)D, RIA	categorical, 20 ng/mL threshold	12 months	hypertension and decreased bone mineral density
Quraishi *et al*. [21]	gastric bypass	retrospective	47	770	no	25(OH)D, not reported	categorical, 10, 20 and 30 ng/mL thresholds compared	variable	surgical site, and hospital-acquired (CRUTI, pneumonia, bacteremia) infections
Ducloux *et al*. [22]	kidney transplant	retrospective	46	363	no	25(OH)D, not reported	categorical, <10, 10–32, > 32 ng/mL thresholds	24 months	cancer
Falkiewiscz *et a*l. [23]	kidney transplant	prospective, observational	43	90	no	1,25-(OH)_2_D, RIA	categorical, 15 pg/mL threshold	24 months	delayed graft function and graft loss
Kim *et al*. [24]	kidney transplant	retrospective	40	106	no	25(OH)D, RIA	categorical, 10 ng/mL threshold	36 months	decreased graft function, and biopsy proven acute rejection
Bienaime *et al*. [25]	kidney transplant	prospective, observational	48	634	no	25(OH)D and 1,25-(OH)_2_D, not reported	continuous	12 months	decreased graft function, interstitial fibrosis and tubular atrophy
Lee *et al*. [26]	kidney transplant	retrospective	52	351	no	25(OH)D, RIA	categorical, 20 ng/mL threshold	12 months	acute cellular rejection
Bitetto *et al*. [27]	liver transplant	retrospective	55	133	no	25(OH)D, RIA	categorical, <5, 5–12.5, > 12.5 ng/mL thresholds	8 months	acute cellular rejection
Lowery *et al*. [28]	lung transplant	retrospective	51	102	no	25(OH)D, RIA	categorical, 30 ng/mL threshold	12 months	acute cellular rejection, bacterial, viral, fungal infection, increased one year mortality
Bashutski *et al.*, [29]	periodontal open flap debridement	prospective, randomized trial	48	40	no	25(OH)D, not reported	categorical, 20 ng/mL threshold	12 months	loss of clinical attachment, increased probing depth, decreased bony defect resolution
Turan *et al*. [30]	non-cardiac surgery	retrospective	58	3509	yes	25(OH)D, not reported	continuous, and by quintile	to discharge	increased risk for composite in-hospital mortality, serious infections and cardiovascular events
Zitterman *et al*. [31]	cardiac transplant	prospective, observational	58	171	no	25(OH)D and 1,25-(OH)_2_D, not reported	categorical, 10 ng/mL threshold	12 months	Increased one year mortality
Borgermann *et al*. [32]	coronary bypass graft, valve replacement	prospective, observational	70	59	no	25(OH)D, RIA, and 1,25-(OH)_2_D, ELISA	continuous	30 days	increased risk for composite in-hospital mortality, myocardial infarction, low cardiac output syndrome, infection, and stroke, decreased glomerular filtration rate
Zittermann *et al.* [33]	cardiac transplant and non-transplant cardiac surgery	prospective, observational	not reported	208	no	25(OH)D, RIA, and 1,25-(OH)_2_D, ELISA	continuous	30 days	decreased glomerular filtration rate
Turan *et al*. [34]	cardiac surgery	retrospective	not reported	426	yes	25(OH)D, not reported	continuous	30 days	none
Zittermann *et al*. [35]	non-transplant cardiac surgery	prospective, observational	70	4418	no	25(OH)D, RIA	categorical, <12, 12–20, 20–30, 30–40, >40 ng/mL thresholds	12 months	increased risk of in-hospital mortality, myocardial infarction, low cardiac output syndrome, stroke, 6 and 12 month mortality; U-shaped risk for prolonged ventilatory support and intensive care unit (ICU) stay
Zittermann *et al*. [36]	non-transplant cardiac surgery	prospective, observational	70	3371	no	25(OH)D, RIA, and 1,25-(OH)_2_D, LC-MS/MS	categorical, <12, 12–20, 20–30, 30–40, >40 ng/mL thresholds, and by quintile	to discharge	increased risk of in-hospital mortality, myocardial infarction, low cardiac output syndrome, stroke;
Sriram *et al*. [37]	cardiac surgery	prospective	59	64	no	LC-MS/MS	continuous and categorical	to discharge	prolonged hospital length of stay
Graham *et al*. [38]	cardiac bypass surgery	prospective, observational	9 days	70	no	25(OH)D, RIA	categorical, 20 ng/mL threshold	to discharge	increased inotrope requirement
McNally *et al*. [39]	congential heart disease surgery	prospective, observational and retrospective	6 months	58	no	25(OH)D, LC-MS/MS	continuous, and categorical, 10 ng/mL threshold	to discharge	increased fluid and inotrope requirement, and intubation duration

RIA: radioimmunoassay; CIA: chemoluminescent immunoassay; HPLC: high pressure liquid chromatography; LC-MS/MS: liquid chromatography tandem mass spectrometry; ELISA: enzyme-linked immunosorbent assay.

Five publications observed no significant difference in the incidence of adverse outcomes between groups with lower and higher vitamin D status [11,14,15,17,34]. However, in none is it clear that no difference truly exists, or if design and conduct constraints preclude detection of a correlation. For example, Jansen *et al*. concede “missing data could be responsible for lack of power to show any significance at all”. [15]. Similarly, Unnanuntana conclude “Considering that our definition of vitamin D deficiency was determined at higher serum levels than previously reported, this could potentially explain the apparent lack of association between vitamin D status and the attainment of functional milestones”. [11]. In accounting for failure to observe an association between perioperative vitamin D concentrations and cardiac surgery outcomes in a retrospective analysis of an administrative database, Turan *et al*. suggest that outcomes after the cardiovascular procedures that they examined may be “overwhelmingly determined by other baseline and surgical factors” [34]. Sixteen manuscripts describe the lack of statistical significance of one or more associations between vitamin D status and a diversity of secondary outcomes. One report suggests a U-shaped relationship between serum 25(OH)D concentrations and adverse cardiovascular outcomes after cardiac surgery at levels above 40 ng/mL [35]. The correlation was later refuted in a follow-up investigation by the same investigators [36]. No other deleterious associations with high vitamin D status were disclosed.

Vitamin D status was first measured in the interval from 2 weeks before surgery to immediately after anesthetic induction in 22 studies. In 2 reports, vitamin D status was first tested up to 2 weeks after surgery [23,33]. Vitamin D status was first determined in the interval from 2 weeks before to 12 weeks after surgery in 2 investigations [25,26]. In 4 reports, vitamin D status was assessed in the interval from 12 weeks before surgery to 4 weeks after surgery [9,21,20,34]. Lowery *et al*. examined vitamin D status in the interval from 12 weeks before to 12 weeks after surgery [28]. In 13 reports, 25(OH)D was measured by radioimmunoassay (RIA), in 5 reports by liquid chromatography and tandem mass spectrometry (LC-MS/MS), in 1 report by high pressure liquid chromatography (HPLC), and in 2 reports by chemoluminescent immunoassay (CIA). Five teams of investigators failed to indicate their methods of measuring serum 25(OH)D. Two reports provide results from both 25(OH)D by RIA and 1,25(OH)_2_D by enzyme-linked immunosorbent assay (ELISA), 1 from both 25(OH)D by RIA and 1,25(OH)_2_D by LC-MS/MS, 2 from both 25(OH)D by RIA and 1,25 (OH)_2_D by unreported methods, and 1 from 1,25(OH)_2_D alone using RIA.

In 9 manuscripts, vitamin D status is analyzed and reported as a continuous variable. Three of the 9 also analyze and report vitamin D status as a categorical variable, or as a quintile. In 22 investigations, vitamin D status was solely analyzed and reported as a categorical (*i.e.*, dichotomized or trichotomized) variable. 25(OH)D thresholds for the lowest analyzed cutoff vary widely from 5 ng/mL (1 report), 10 ng/mL (4 reports), 12 ng/mL (2 reports), 16 ng/mL (2 reports), 20 ng/mL (7 reports), to 30 ng/mL (4 reports) between studies (Table 1). The number and proportion of participants in categories defined and analyzed by cutoff values were broadly unequal between categories in 19 of the 22 investigations. Twenty-six of 31 investigations failed to perform a power analysis to assure that overall sample sizes were adequate to detect a significant difference based on estimated variances around anticipated sample means. Twenty-three of 31 investigations performed one or more multivariate analyses [9,10,12,13,16-18,21,22,24-27,30-39].

## Discussion

The aim of the present contribution is to provide a systematic review of investigations published in peer-reviewed periodicals that compare risks for diverse post-surgical outcomes with perioperative measures of vitamin D status based on a search designed to include any surgery in both adult and pediatric patients. Surprisingly, no publications that satisfy inclusion criteria were published before 2008, although validated indices of vitamin D status and quantifiable measures of post-operative outcomes have been available for decades. The absence of earlier manuscripts most probably reflects failure of clinicians and investigators to recognize the potential for adverse, extra-skeletal consequences of low vitamin D status in acute care settings as a research question of interest. Correlation of vitamin D status with the risk and severity of over 130 chronic non-skeletal conditions including death due to cardiovascular disease, cancer and other causes is contentious [40,41]. Compared to susceptibilities that may require decades to evolve, a first strength of reports incorporated in the present review is that adverse outcomes are linked in time to a surgical intervention that is shared between participants with lower and higher vitamin D status. A second strength is that medical and surgical records in the interval surrounding surgery are rich with objective data including physical examinations, laboratory values, past medical histories and the like, so that background variables including co-morbidities, inter-current medications, and body mass indices that influence markers of vitamin D status as well as surgical outcomes may be closely matched, and results that may arise from reverse causation otherwise controlled. A further strength is that evidence that is evenly divided between prospective and retrospective study designs points to shared observations. Statistically significant and clinically important correlations of adverse outcomes after surgery with low vitamin D status in 26 of 31 manuscripts suggest that vitamin D status is a predictor of extra-skeletal disorders for which predispositions may be diagnosed and deficiencies corrected prior to surgical procedures. In turn, publication of 5 reports that do not identify statistically significant correlations, and lack of statistical significance of one or more associations between vitamin D status and a diversity of secondary outcomes in 17 of 31 manuscripts, implies that positive publication bias alone is insufficient to account for an overall association.

Another surprising feature of the present review is the diversity, severity and magnitude of increased risks for detrimental outcomes that span suboptimal surgical outcomes to grievous postoperative complications including surgical site and hospital-acquired infections, graft failure, cancer, myocardial infarction, low cardiac output syndrome, stroke, ICU and hospital length of stay, and in-hospital and one-year mortality after surgery. While not the objective of the reviewed publications, virtually all inquiries confirm a very high prevalence of vitamin D deficiency at the time of surgery in view of 25(OH)D levels recommended by the Institute of Medicine (20 ng/mL) and The Endocrine Society (30 ng/mL) to maintain skeletal health [3,42]. Several studies suggest that vitamin D status *at the time of surgery* is the most relevant predictor of long term outcomes compared to vitamin D status in days to weeks after surgery, and that minimal benefits can be gained by supplementation at the time of surgery or thereafter [24,29]. Survey of publications summarized in Table 1 as a whole demonstrates that most patients arriving for surgery are malnourished, and reveals that the patient safety and public health consequences of malnutrition may be serious.

Inspection of Table 1 further highlights sizeable gaps in the quality and quantity of evidence of potential utility in guiding clinical decisions. Standardization of measures chosen for determination of vitamin D status as an independent variable must be a top priority of future investigations seeking data with acceptable analytical validity. Endocrine Society Task Force guidelines recommend measurement of circulating 25(OH)D rather than 1,25(OH)_2_D to assess vitamin D status, or both, but disparages analysis of 1,25(OH)_2_D alone [3]. 25(OH)D assays using LC-MS/MS methods calibrated with National Institutes of Standards and Technology (NIST) standards are to be preferred over RIA- and ELISA-based technologies that may over-estimate true 25(OH)D concentrations [43]. Because precision and predictive power differ between kits and manufacturers, when RIA- and ELISA-based methods are chosen, investigators must provide the source, assure calibration with consensus standards, and cite references of comparative accuracy. Incorporation of plasma and urine measures of vitamin D binding protein (VDP) and free 25(OH)D is encouraged in order to interpret fluctuations in 25(OH)D levels that may arise from pre-operative and post-operative fluid management, nutrition, health status and other factors known to shift in the perioperative setting [37,44]. The plasma half-life of 25(OH)D is about 3 weeks. 25(OH)D is 80%-90% bound to VDP, with its half-life measured in days. For these reasons, a molar ratio of 25(OH)D to VDP concentrations may provide a useful index of vitamin D biological activity of value in longitudinal study designs. Given 25(OH)D and VDP kinetics, determination of vitamin D status in the perioperative interval should be further standardized to include at least one measure in the time period 2 weeks before surgery to incision. In addition, funding sources and journal editors must expect strong justifications for not acquiring serial vitamin D assays at the time of scheduled post-operative outcome measures in order to fill gaps in published data.

Multiple opportunities are available to improve the clinical validity of presumptive correlations between perioperative vitamin D status and post-operative outcomes. A minority of investigations in the present review properly report and analyze vitamin D status as a continuous variable. Thresholds and cutoffs that compress vitamin D status into 2 or 3 categories based on skeletal outcomes are hotly debated among experts [45]. No threshold levels have been validated for extra-skeletal outcomes. Whereas minimal daily requirements and levels of vitamin D to avoid traits of musculoskeletal deficiency fall most reasonably in the range of 20 to 30 ng/mL, 25(OH)D concentrations necessary for optimal health, and 25(OH)D concentrations necessary for *optimal health during recovery from surgery and critical care*, are knowable but unknown [46]. Clearly, optimal levels of serum 25(OH)D that may differ between patients, surgeries and outcomes must be sought, validated and replicated. A key path toward resolution of thresholds with clinical validity will be to first correlate 25(OH)D as a continuous variable to outcomes of interest. Analysis and reporting of vitamin D status as a categorical variable may be considered as a supplement to analysis of 25(OH)D concentration as a continuous variable of potential use to frame relationships with collateral data sets and arbitrary definitions (*e.g.*, “deficient”, “inadequate”, “sufficient”, “normal”), but categorical variables must not be considered a substitute for more rigorous and comprehensive methods. Uniform descriptive and inferential statistical methods and reports appropriate for analysis of continuous variables will enable investigations to be compared with one another without loss of data that is inevitable when categorical variables above and below an arbitrary threshold are tested and described alone. High levels of clinical validity further rely on standardization of outcome definitions and measures as dependent variables within and between surgical specialties and sub-specialties coupled to standardized evaluation time frames.

Manuscripts included in the present review illustrate that superior experimental designs are required to disentangle potential correlations between perioperative vitamin D status and complications after surgery. Because testing for and correcting vitamin D deficiency is low in cost, safe and effective, inappropriate and inadequate designs, *i.e.*, underpowered “pilot” studies, are a temptation to be avoided. Population sample sizes of the present data are often too small to permit ironclad conclusions on the basis of a single publication. Failure to perform a power analysis for a primary outcome in 26 of 31 reports is an error that must be avoided by investigators, and that must not be tolerated by funding agencies, editors, reviewers and readers. Interpretation of results between studies relies on explicit provision of inclusion and exclusion criteria, and matching of background variables that modify assays of vitamin D status and outcome measures. No investigation to the present has enrolled matched participants within no-surgery arms. Most studies are confined to a single center despite internationally standardized measures of vitamin D status and outcome specific measures. To circumvent bias, unmeasured cofounders and insufficient power, investigations directed to interrogation of administrative or other databases using propensity score matching must satisfy requirements that are often skirted [47]. Future prospective trial designs in which vitamin D status is manipulated in the perioperative interval must measure concentrations at baseline before surgery, and then administer vitamin D analogs guided by pre-set target concentrations rather than by fixed doses. Investigators should consider experimental designs that are *a priori* compliant with Newcastle-Ottawa Scale guidelines for observational studies, and with the Cochrane Risk Assessment Tool for randomized controlled trials in order that future evidence across studies for diverse outcomes may be meaningfully compared [48,49].

Defects in statistical methods and reporting are prevalent in the manuscripts of the present review. Investigators should target enrollment that satisfies normal distributions of both vitamin D status and outcome measures. When non-parametric inferential methods and data transformations are employed, publications should declare which assumptions for parametric analysis have been violated. If odds ratios and hazard ratios are reported, authors must make it simple for the reader to locate the number and proportion of patients with a given outcome that may have contributed to a statistically significant association, and provide confidence intervals to support comparisons with the present and future data of others. In articles of the present review, authors are inconsistent in using multivariate analyses to correct for confounders (*e.g.*, age, gender, body mass index), and in correcting for multiple comparisons that encroach on available statistical power. Statistical methods that test for the relative effect sizes of multiple outcomes should be employed whenever appropriate [50].

The present systematic review underscores the need for replication and extension of published results, as well as the need for future investigations of vitamin D status as a predictor of other outcomes after other types of surgery and anesthesia. For example, vitamin D deficiency increases the risk of cognitive decline and dementia in older adults, but the acute and chronic effects of low vitamin D status at the time of surgery on postoperative delirium, cognitive dysfunction, and the onset and progression of dementia after surgery are unknown [51,52]. Enrollment of a much broader diversity of age groups from infancy to senescence, co-morbidities, and ethnicities together with cohorts matched for gender and gravidity is compelling in view of presently available data. Vitamin D synthetic and metabolic pathways are highly polymorphic. Shared serum levels of free 25(OH)D may therefore carry distinct health implications in populations with divergent pharmacokinetic and pharmacodynamic genetic backgrounds. To this end, addition of molecular biomarkers including genomics, epigenomics, and assays of expressed damage associated patterns (DAMPs) (*e.g.*, by mass spectrometry of serum samples) to perioperative vitamin D protocols is clinically and scientifically warranted, and very likely to be informative in comparisons of extreme phenotypes *e.g.*, of bottom vs. top quintiles of vitamin D status and specific outcomes [53]. Of note, hypovitaminosis D may itself be a marker for deficiencies of other vitamins, nutrients and co-factors in the perioperative interval, with consequences that may also have been overlooked after surgery. As knowledge of the effects of malnutrition at the time of surgery expands, submission and publication of well-designed and conducted studies with negative results will be particularly crucial as a counterweight to positive publication bias.

While awaiting publication of data with greater breadth, depth and rigor, we propose that the contents of the present systematic review comprise a message of present day clinical relevance. There is no question that a large proportion of patients undergo surgery and anesthesia in developed and developing nations with moderate to profound levels of vitamin D deficiency. Publications collected and reviewed in the present contribution provide ample evidence that many patients are at substantially heightened risk for calamitous outcomes by virtue of their low vitamin D status at the time of surgery. Nevertheless, contemporary practice is not to test for, or to supplement, vitamin D concentrations before surgical procedures. We propose, to the contrary, that manuscripts reviewed here provide sufficient evidence to shift the burden of proof to those who believe that performing elective surgery on vitamin D deficient patients comports with the highest standards of patient safety and public health. Until such data is available, and in consideration of the low cost, safety and efficacy of supplementation of a nutrient (*i.e.*, not a drug, foreign chemical, or blood product), we further propose that evidence is sufficient at present to support testing and supplementation to target levels as a practical default. We contend that learning whether it is safe to deviate far from ancestral levels of vitamin D in patients facing the trauma of surgery, and the demands of healing, is an overarching question, and that until this answer is in hand measurement and supplementation as indicated is preferred to the no-action approach of the *status quo* [54].

We are aware of several limitations of the present systematic review. A first is the possibility that one or more publications may have eluded our scrutiny. A second limitation is that the heterogeneity of patients, surgeries, methods of measuring vitamin D status and postoperative outcomes, statistical inference and reporting precludes formal analysis of the quality of evidence across studies, and combination of the studies for meta-analysis at present [55]. A third limitation is that none of the manuscripts were configured to illuminate mechanisms of the interaction between perioperative vitamin D status and outcomes after surgery. In the absence of well-settled mechanisms, close matching of preoperative health status and co-morbidities at the time of surgery, and observations made in phase with a specific surgical intervention, controls but does not fully eliminate the possibility that vitamin D status is a bystander or proxy for ill health and reverse causation. Randomized controlled trials are required to balance bias and unmeasured confounders, however evidence from the present manuscripts suggests that IRB approval of placebo-based protocols, and voluntary enrollment in placebo cohorts, may not be trivial undertakings if full disclosure of present data is furnished to candidate participants.

## Conclusions

In conclusion, the present systematic review establishes that a large majority of prospective and retrospective investigations report that low perioperative vitamin D status is associated with a diversity of adverse outcomes after surgery with statistical significance and clinical importance. Taken individually, manuscripts included in the present contribution must be viewed with caution in light of methods that may over-estimate vitamin D status, that are confined to categorical analysis and reporting, and that may comprise heterogeneity that limits comparisons between studies. Taken together, manuscripts included in the present contribution provide evidence that an opportunity for substantial improvement in patient safety and public health is expedient. Practice improvements of comparable potential magnitude with negligible cost and exemplary safety and efficacy are rare, and raise deliberation of the ethical implications of choosing not to test for, and not to treat, low vitamin D status in advance of surgery.
